# Hippocampal volume change following ECT is mediated by rs699947 in the promotor region of *VEGF*

**DOI:** 10.1038/s41398-019-0530-6

**Published:** 2019-08-20

**Authors:** Maarten J. A. Van Den Bossche, Louise Emsell, Annemiek Dols, Kristof Vansteelandt, François-Laurent De Winter, Jan Van den Stock, Pascal Sienaert, Max L. Stek, Filip Bouckaert, Mathieu Vandenbulcke

**Affiliations:** 1 Department of Geriatric Psychiatry, University Psychiatric Center KU Leuven, Leuven, Belgium; 2 Center for Neuropsychiatry, Research Group Psychiatry, Department of Neurosciences, KU Leuven, Leuven, Belgium; 3 Department of Old Age Psychiatry, GGZ inGeest/VU University Medical Center, Amsterdam Public Health Research Institute, Amsterdam Neuroscience, Amsterdam, the Netherlands; 4 Department of Adult Psychiatry, University Psychiatric Center KU Leuven, Leuven, Belgium

**Keywords:** Personalized medicine, Molecular neuroscience, Depression, Prognostic markers, Clinical genetics

## Abstract

Several studies have shown that electroconvulsive therapy (ECT) results in increased hippocampal volume. It is likely that a multitude of mechanisms including neurogenesis, gliogenesis, synaptogenesis, angiogenesis, and vasculogenesis contribute to this volume increase. Neurotrophins, like vascular endothelial growth factor (VEGF) and brain-derived neurotrophic factor (BDNF) seem to play a crucial mediating role in several of these mechanisms. We hypothesized that two regulatory SNPs in the *VEGF* and *BDNF* gene influence the changes in hippocampal volume following ECT. We combined genotyping and brain MRI assessment in a sample of older adults suffering from major depressive disorder to test this hypothesis. Our results show an effect of rs699947 (in the promotor region of *VEGF*) on hippocampal volume changes following ECT. However, we did not find a clear effect of rs6265 (in *BDNF*). To the best of our knowledge, this is the first study investigating possible genetic mechanisms involved in hippocampal volume change during ECT treatment.

## Introduction

Electroconvulsive therapy (ECT) is the most effective biological treatment for major depressive disorder^1^. A higher age is associated with a better outcome^2,3^. Despite the proven excellent effectiveness of this treatment, much of the exact working mechanisms of ECT remain largely unknown^4^.

Several studies, both in late-life depression and younger adults have shown that ECT results in an increased hippocampal volume^5,6^. This increase seems to be dose-dependent, with a higher number of ECT sessions resulting in more pronounced enlargement^7^. Several mechanisms may play a role in these hippocampal volume changes. Animal models have shown an electroconvulsive shock (ECS)-related increase in neurogenesis and synapse formation^8–10^, angiogenesis and proliferation of endothelial cells^11–17^ and gliogenesis^18,19^. Human studies have shown changes in regional blood flow^20,21^ and immunological and inflammatory responses following ECT^22^. Rather than one single mechanism, it is more likely that a multitude of mechanisms including neurogenesis, gliogenesis, synaptogenesis, angiogenesis, and vasculogenesis contribute to the increase in hippocampal volume following ECT, as there is a close interconnection between these systems^23^. For example, endothelial cells can influence neurogenesis by secreting factors, which act on neural stem and progenitor cells^15^. An increase in the number of synapses and changes in dendritic structures might be accompanied by growth of both capillaries and glia^24^, perhaps to support increased energy demand of the new synapses^25^.

Neurotrophins, like vascular endothelial growth factor (VEGF) and brain-derived neurotrophic factor (BDNF) seem to play a crucial mediating role in several of these mechanisms that result in hippocampal volume changes. VEGF is the most potent inducer of blood vessel growth^26^. It is also expressed in the brain, and has an important role in promoting neurogenesis, neuronal migration, neuronal survival and axon guidance^27–29^. VEGF has been linked to antidepressant treatment response^30^. An association between low baseline serum VEGF levels and non-response to ECT has been found^31^. In a recent study a moderate negative correlation between the change in VEGF plasma levels and change in depression severity following bitemporal (BT) ECT was identified, however, no other associations between VEGF and mood or responder/remitter status were found^32^.

The expression of VEGF can be regulated by certain single nucleotide polymorphisms (SNPs). The common SNP rs699947 in the promotor region of the *VEGF* gene (also known as 2578 C/A) significantly affects serum levels of VEGF^33^. Moreover, an association between the *VEGF* 2578 C/A polymorphism and treatment-resistant depression was reported^34^. An impact of this polymorphism on total grey and total white matter volume and on total arterial blood volume in the brain was shown^35^.

BDNF is an important neurotrophin and plays a key role in neuronal development and neuroplasticity^36^. BDNF is consistently low in patients with major depression^37^, nevertheless, it lacks diagnostic specificity as it can be reduced in other psychiatric disorders as well^38–41^. In animal studies it was shown that chronic ECS induces hippocampal mossy fiber sprouting, and that the expression of BDNF seems to play a crucial role in the induction of this sprouting^42,43^.

Rs6265 is a widely studied SNP in the *BDNF* gene. As a result of a G(uanine) to A(denine) substitution in the gene at this position, a valine (val) to methionine (met) substitution in the 5’ proregion of the BDNF protein occurs, resulting in a reduced mature BDNF expression in met-carriers^44^. The met-variant is more common in late-life depression^45,46^ and is associated with smaller hippocampal volumes in both patients with major depression and healthy controls^47,48^. Peripheral BDNF levels have shown to increase after ECT in some but not all studies^49^.

We hypothesize that these two regulatory SNPs in two important neurotrophins influence the changes in hippocampal volume during ECT treatment. We combined genotyping and brain MRI assessment in a sample of older adults suffering from major depressive disorder to test this hypothesis.

## Materials and methods

### Subjects and characteristics

We included all subjects that participated in the Mood Disorders in Elderly treated with ECT (MODECT) study^50^ (clinical trial NCT02667353) and from whom genetic material as well as MRI scans pre- and post-ECT were available (61 subjects of total MODECT sample (*n* = 110), University Psychiatric Center KU Leuven, Belgium (*n* = 31) and GGZ inGeest Amsterdam, the Netherlands (*n* = 30)). All subjects were 55 or older and had a DSM-IV diagnosis of major depressive disorder, with or without psychotic features. The diagnosis was made by a psychiatrist and confirmed by the Mini International Neuropsychiatric Interview (MINI)^51^. Exclusion criteria were other major psychiatric illness, a major neurological illness (including Parkinson’s disease, stroke and dementia) and contraindications for MRI. Psychotropic medication was discontinued at least 1 week prior to ECT, or if deemed impossible, kept stable from 6 weeks prior to ECT and during the ECT-course^50^.

Depressive symptoms were monitored 1 week prior to ECT and 1 week after the last ECT using the Montgomery-Åsberg Depression Rating Scale (MADRS)^52^. MADRS scores before and after ECT were available for 58 subjects.

All patients provided written informed consent. The ethical committees of the two centers approved this study.

### ECT procedure

ECT was administered twice a week with a constant-current brief pulse device (Thymaton System IV, Somatics, IL, USA). Anesthesia was achieved with intravenous administration of etomidate (0.2 mg/kg) and succinylcholine (1 mg/kg). Seizures were monitored to ensure adequate duration and quality. All subjects were treated with right unilateral (RUL) ECT with stimulus intensity 6 times the initial seizure threshold (ST), as determined by empirical dose titration at the first treatment, until remission. Subjects who failed to respond to RUL after the sixth treatment were switched to bitemporal (BT) ECT (1.5xST)^50^. All patients were treated with brief pulse (0.5–1.0 ms) ECT.

### MRI procedure

Structural MRI was performed 1 week prior to ECT and 1 week after the last ECT. High resolution 3D T1-weighted images were acquired using an 8-channel head-coil with a 3D turbo field echo sequence on a 3T Philips Intera scanner in Leuven and on a 3T GE Signa HDxt scanner in Amsterdam (TR = 9.6 s, TE = 4.6 s, flip angle = 8°, slice thickness = 1.2 mm, in-plane voxel-size = 0.98 × 1.2 mm^3^, 182 slices, acquisition time = 383 s).

### Hippocampal volume

A trained rater blinded to time-point manually delineated the hippocampus in native space following an initial automatic segmentation step^53^. Manual editing was performed using ITK-SNAP version 2.4 (http://www.itksnap.org/pmwiki/pmwiki.php) in accordance with the HarP guidelines^54^. Hippocampal volumes were normalized based on total brain volume^55^, using a standard approach^56^.

### Genotyping of polymorphisms

Genomic DNA was extracted from peripheral blood using standard methods. The SNP rs6265 in *BDNF* and rs699947 in the promotor region of *VEGF* were genotyped by a real-time polymerase chain reaction with melting curve analysis using a LichtCycler 480 II instrument (Roche, Penzberg, Germany). For each SNP a LightSNiP kit (TIB Molbiol, Berlin, Germany) was used with pre-mixed primer and probes specific for each SNP. Melting temperatures were called using Lightcycler 480 Software 1.5.1 (Roche, Penzberg, Germany).

### Statistical analyses

Statistical analyses were performed using SPSS Statistics 25 (IBM, Chicago, IL, USA).

Differences in demographic or clinical variables (age, sex, RUL and BT ECT, late onset of depression, baseline hippocampal volume) between rs699947 and rs6265 genotype groups were analyzed using a *t*-test (for age and baseline hippocampal volume) or chi-square test (for sex, RUL/BT ECT, late onset of depression).

Differences in hippocampal volume and MADRS scores pre- and post- ECT were analyzed with a paired samples *t*-test.

Association between rs699947 and rs6265 genotypes and changes in normalized total hippocampal volume and association between SNP genotypes and change in MADRS scores, were analyzed using one-way ANOVA, followed by one-way analysis of covariance (ANCOVA) to control for number of ECTs. Kolmogorov–Smirnov test showed normal distribution of residuals and Brown–Forsythe test showed homogeneity of variance across different genotype groups for both SNPs. Bonferroni correction was applied for multiple testing on the number of SNPs tested ( = 2). As our group has identified lateralization of volume changes following ECT^57^, post-hoc we also analyzed changes in right and left hippocampal volume separately.

Association between hippocampal volume changes and change in MADRS scores was analyzed using linear regression. Assumptions for linear regression were met.

The significance level for statistical tests was set at *p* < 0.05.

## Results

### Demographic and clinical characteristics

The mean age of the 61 subjects included was 72.3 (range 55–90; *SD* = 8.6) with 37 female and 24 male subjects. 17 subjects, who failed to respond initially to RUL ECT were switched after the sixth session to BT ECT. 29 subjects had a first depressive episode after the age of 55 (“late onset”). The distribution of the genotypes were: for rs699947 (SNP in the promotor region of *VEGF*) C/C in 15 subjects, C/A in 29 subjects and A/A in 17 subjects; for rs6265 (SNP in *BDNF*) G/G in 30 subjects, G/A in 25 subjects, A/A in 6 subjects. There were no significant group differences, in sex, age, RUL/BT ECT, onset of depression and baseline hippocampal volumes, between the 3 genotype groups of both SNPs.

### Association between SNP genotypes and change in hippocampal volume

There was a significant increase in normalized total hippocampal volume following ECT (pre-ECT: *M* = 6794.18 mm^3^, *SD* = 767.98; post-ECT: *M* = 6978.56 mm^3^, *SD* = 864.55; *t*_(60)_ = 4.344, *p* *=* 0.000055).

**Fig. 1:**
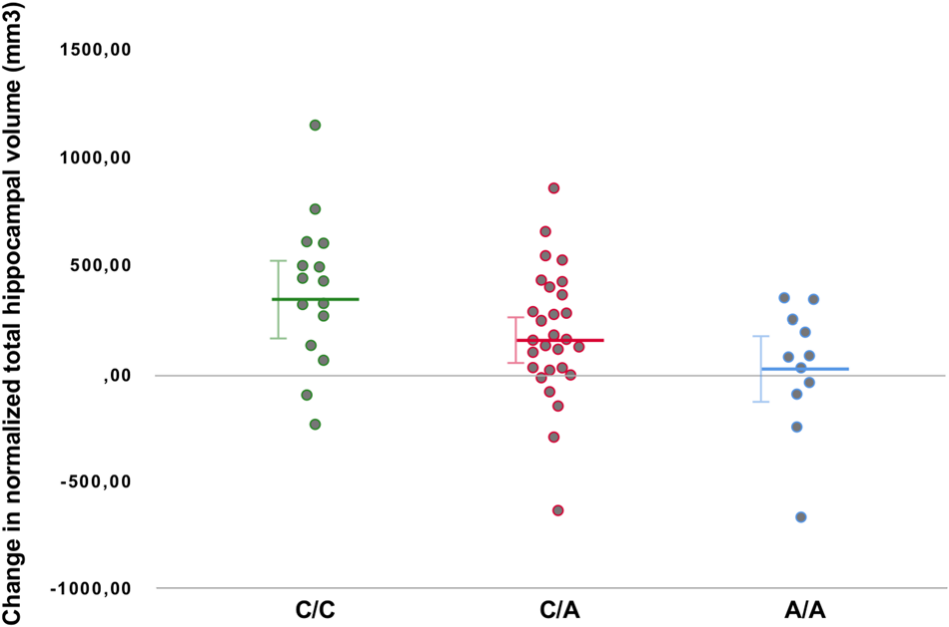
Effect of rs699947 genotype on normalized total hippocampal volume change. Change in normalized total hippocampal volume (in mm^3^) following ECT is plotted for the different genotypes of rs699947 (SNP in the promotor region of *VEGF*). Lines mark means and 95% confidence intervals

A significant association was observed between rs699947 (SNP in the promotor region of *VEGF*) genotype and change in normalized total hippocampal volume (*F*_(2,58)_ = 4.799, *p* = 0.012). When corrected for number of ECT sessions this association remained significant (*F*_(2,57)_ = 3.516, *p* = 0.021). After Bonferroni correction for testing two SNPs, the association remained significant (*p* < 0.025). The effect size of the association between rs699947 genotype and changes in normalized total hippocampal volume was *η*^*2*^ = 0.14, and after correction for number of ECT sessions *η*^*2*^ = 0.16. Post hoc pairwise comparisons using the Tukey HSD test revealed that normalized total hippocampal volume increased significantly less following ECT (*p* = 0.009) in the A/A genotype group (*M* = 33.00 mm^3^, *SD* = 315.57), compared to the C/C genotype group (*M* = 374.80 mm^3^, *SD* = 344.42). Increase in normalized total hippocampal volume in the C/A genotype group was intermediate (*M* = 174.62 mm^3^, *SD* = 293.01) between, but not significantly different from, the C/C and A/A genotype group (*p* = 0.118 and *p* = 0.306, respectively). Post hoc comparison of the estimated marginal means after ANCOVA (with the number of ECT sessions as covariate) with Bonferroni correction again showed significantly lower increase of normalized total hippocampal volume in the A/A group compared to the C/C group (*p* = 0.013) and increase in the C/A group being intermediate between, but not statistically significantly different from, the C/C and A/A group (*p* = 0.142 and *p* = 0.545, respectively). The association between rs699947 genotype and normalized total hippocampal volume change is illustrated in Fig. 1.

We could not find a significant association between rs6265 (SNP in *BDNF*) and change in normalized total hippocampal volume (*F*_(2,58)_ = 3.066, *p* = 0.054). When corrected for number of ECT sessions this association weakened further (*F*_(2,57__)_ = 2.263, *p* = 0.091).

As the volume increase was more pronounced on the right side (pre-ECT: *M* = 3431.24 mm^3^, *SD* *=* 411.80; post-ECT: *M* = 3553.68 mm^3^, *SD* = 465.23; *t*_(60)_ = 5.095, *p* = 0.000004) versus the left side (pre-ECT: *M* = 3362.94 mm^3^, *SD* = 400.62; post-ECT: *M* = 3424.88 mm^3^, *SD* = 428.39; *t*_(60__)_ = 2.373, *p* = 0.021), we then looked at the association with volume changes of the right and left hippocampus separately. For rs699947 we found a strong association with changes in normalized right hippocampal volume (*F*_(2,58)_ = 6.132, *p* = 0.004), which remained significant after correction for number of ECT sessions (*F*_(2,57)_ = 4.043, *p* = 0.011), but no significant association with left hippocampal volume changes (*F*_(2,58)_ = 1.943, *p* = 0.152; after correction for number of ECT sessions: *F*_(2,57)_ = 2.393, *p* = 0.078). The effect size of the association between rs699947 genotype and changes in normalized right hippocampal volume was *η*^*2*^ = 0.18, and after correction for number of ECT sessions remained *η*^*2*^ = 0.18. Post hoc pairwise comparisons using the Tukey HSD test revealed that normalized right hippocampal volume increased significantly less (*p* = 0.003) in the A/A genotype group (*M* = 12.29 mm^3^, *SD* = 194.88), compared to the C/C genotype group (*M* = 225.40 mm^3^, *SD* = 198.17). Increase in normalized right hippocampal volume in the C/A genotype group was intermediate (*M* = 133.76 mm^3^, *SD* = 144.84) between, but not significantly different from, the C/C and A/A genotype group (*p* = 0.229 and *p* = 0.065, respectively). Post hoc comparison of the estimated marginal means after ANCOVA (with the number of ECT sessions as covariate) with Bonferroni correction again showed significantly lower increase of normalized right hippocampal volume in the A/A group compared to the C/C group (*p* = 0.003) and increase in the C/A group being intermediate between, but not statistically significantly different from, the C/C and A/A group (*p* = 0.317 and *p* = 0.078, respectively).

For rs6265 we did not find an association between changes in normalized right (*F*_(2,58)_ = 1.799, *p* = 0.175; after correction for number of ECT sessions: *F*_(2,57__)_ = 1.199, *p* = 0.318) nor left (*F*_(2,57__)_ = 2.493, *p* = 0.091; after correction for number of ECT sessions: *F*_(2,57__)_ = 2.546, *p* = 0.065) hippocampal volume.

### Clinical outcome and association with SNP genotypes and hippocampal volume changes

There was a significant decrease in MADRS scores following ECT (pre-ECT: *M* = 34.31, *SD* = 9.40; post-ECT: *M* = 9.93, *SD* = 9.83; *t*_(57)_ = 14.806, *p* *=* 3.45 × 10^−21^).

No association between genotypes of the 2 SNPs and change in MADRS score prior versus after ECT could be found (for rs699947: *F*_(2,55__)_ = 0.262, *p* = 0.770; after correction for number of ECT sessions *F*_(2,53)_ = 0.778, *p* = 0.545; for rs6265: *F*_(2,55)_ = 0.166, *p* = 0.847; after correction for number of ECT sessions *F*_(2,53__)_ = 0.717, *p* = 0.584).

Simple linear regression showed that changes in normalized hippocampal volumes did not significantly predict change in MADRS score (for total hippocampal volume *R*^2^ *=* 0.001, *F*_(1,56)_ *=* 0.042, *p* *=* 0.838; for right hippocampal volume: *R*^2^ = 0.0001, *F*_(1,56)_ *=* 0.007, *p* *=* 0.933; for left hippocampal volume: *R*^2^ = 0.003, *F*_(1,56)_ *=* 0.165, *p* *=* 0.686).

## Discussion

To the best of our knowledge, this is the first study investigating possible genetic mechanisms involved in hippocampal volume change following ECT.

In line with our hypothesis, our results show an effect of rs699947 genotype on hippocampal volume changes during ECT treatment. Patients with more C alleles tend to have more hippocampal volume increase. The C allele is regarded as the “wild-type” allele, and it was suggested previously that C-carriers have higher VEGF expression than non-carriers^33^.

Animal models of ECT (ECS) have shown upregulation of VEGF and this upregulation was most prominent in the dentate gyrus of the hippocampus^58^. Interestingly, several recent studies highlighted that ECT seems to consistently lead to volume increase in the dentate gyrus subfield^59–61^. It was demonstrated before that VEGF-signaling is necessary and sufficient for ECS-related induction of quiescent neural progenitor cell proliferation in rats^16^. VEGF expression strongly stimulates angiogenesis. VEGF is known to also increase proliferation of other cells like neurons, astroglia and endothelial cells^27^. An animal study showed that VEGF is an essential mediator in restoration of neurogenesis by electroconvulsive seizure after irradiation exposure^17^. Considering these previous findings, our results suggest that the more C alleles at rs699947, the higher the expression of VEGF during ECT treatment, which in turn leads to angiogenesis and neurogenesis, thus leading to more increase in hippocampal volume.

The effect of rs699947 genotype on hippocampal volume change following ECT seems to be largest on the right hippocampus. This is probably due to all patients initially receiving RUL ECT. The changes in total hippocampal volume during ECT in this sample are mainly due to changes in the volume of the right hippocampus. This lateralization could be explained by higher electric field/exposure leading to more structural changes^62^.

Contrary to our hypothesis, we did not find a clear effect of the rs6265 (in *BDNF*) genotype on hippocampal volume changes.

The association of the BDNF Val66Met polymorphism with major depressive disorder remains unclear^63^. In a previous study in a geriatric population no significant association between the BDNF Val66Met polymorphism and hippocampal volume or function was found^64^. The authors hypothesized that other factors may have a stronger effect on hippocampal structure in older individuals and that the association between the Val66Met polymorphism and geriatric depression is mediated through other mechanisms. Our group and other groups also found no significant changes in mean serum BDNF levels following ECT treatment^65–69^. On the other hand it is possible that our sample was too small to detect an effect, with a low number of patients with the A/A genotype (*n* = 6).

In line with what we reported in the larger MODECT study^69^, we did not find an association between hippocampal volume change and change in MADRS score following ECT in this sample. However, some studies did establish a positive correlation between hippocampal volume change and clinical outcome^61,70,71^. Several other studies did not find this effect or identified even a negative correlation^7,69,72–75^. It is possible that hippocampal volume increase following ECT is related more to the electrical current or to the seizure aspect of the treatment, rather than to its therapeutic effects^7^. Larger studies are therefore needed to elucidate the clinical effects of hippocampal volume change following ECT and the role of genetic mechanisms in mediating these effects.

To conclude, our study provides first-in-human evidence that *VEGF* genotype is involved in structural brain changes following ECT and warrants further research into the role of genetic mediators of neural effects induced by brain stimulation techniques.
